# Detection of Seagrass Distribution Changes from 1991 to 2006 in Xincun Bay, Hainan, with Satellite Remote Sensing

**DOI:** 10.3390/s90200830

**Published:** 2009-02-05

**Authors:** Dingtian Yang, Chaoyu Yang

**Affiliations:** LED, South China Sea Institute of Oceanology, Chinese Academy of Sciences, Guangzhou, P.R. China, 510301

**Keywords:** Seagrass distribution change, remote sensing, Xincun bay

## Abstract

Seagrass distribution is a very important index for costal management and protection. Seagrass distribution changes can be used as indexes to analyze the reasons for the changes. In this paper, *in situ* hyperspectral observation and satellite images of QuickBird, CBERS (China Brazil Earth Resources Satellite data) and Landsat data were used to retrieve bio-optical models and seagrass (*Enhalus acoroides, Thalassia hemperichii*) distribution in Xincun Bay, Hainan province, and seagrass distribution changes from 1991 to 2006 were analyzed. Hyperspectral results showed that the spectral bands at 555, 635, 650 and 675 nm are sensitive to leaf area index (LAI). Seagrass detection with QuickBird was more accurate than that with Landsat TM and CBERS; five classes could be classified clearly and used as correction for seagrass remote sensing data from Landsat TM and CBERS. In order to better describe seagrass distribution changes, the seagrass distribution area was divided as three regions: region A connected with region B in 1991, however it separated in 1999 and was wholly separated in 2001; seagrass in region C shrank gradually and could not be detected in 2006. Analysis of the reasons for seagrass reduction indicated it was mainly affected by aquaculture and typhoons and in recent years, by land use changes.

## 1. Introduction

The importance of seagrass in coastal ecosystems had drawn more and more attention these days, as it can provide food and shelter for fish, and also keep the coastal ecological system healthy and protect dams from huge waves. Population increase and industrial development have increased the discharge of polluted water, which has deteriorated the coastal water quality and correspondently reduced seagrass distribution areas. In addition to these reasons, seagrass can also be affected by strong winds and rainfall, aquaculture, propeller currents, etc. In order to protect the vulnerable seagrass ecosystem, six international meetings on seagrass have been held and many researchers in different countries have invested great efforts in detecting the distribution and living state of seagrass.

The traditional method for investigating seagrass distribution and living status was by *in situ* measurement, which is time consuming and expensive. Remote sensing is a very useful method for seagrass detection, for which it is synoptic, repetitive, consistent and cost effective. When seagrass is distributed underwater, visible bands were often used to detect the density and living state, for water absorbs greatly in the red and infra-red spectrum. With the development in recent years of satellite remote sensing, especially high resolution satellite images, such as Landsat MSS, TM, ETM^+^, SPOT, IKONOS and aerial photography, many research projects concerning the distribution and living state of seagrass have been conducted using high resolution satellite remote sensing data.

Landsat data was usually used for detecting seagrass distribution for its cost-effectiveness and relatively higher revisit frequency, and visible bands were regarded as the most useful. Lennon introduced the advantages of satellite remote sensing with Landsat TM data on detection of seagrass in 1989 and regarded red, blue and green as the most useful channels for detecting seagrass distribution [1]. Dahdouh-Guebas [2] also used the blue, red and green band channels of Landsat TM data to map the distribution of seagrass on the Kenyan coast with good results. In order to retrieve seagrass distribution in the wilder Caribbean region, 40 Landsat scenes were processed and three major classes were clearly derived (dense seagrass, medium-sparse seagrass, and a generic “other class”) [3]. When Landsat data was applied for detecting seagrass changes in shallow water from 1988-2002, Dekker found that accuracy was greater than 76% [4]. The main imperfection was that band spectrum coverage was relatively wider and the pixel sizes (20–30 m) are of a similar magnitude to the size of the habitat patches, so this was problematical when Landsat data was used for detecting seagrass in small areas and for distinguishing seagrass species.

The need for precise detection of the living status and distribution of seagrass in small areas led some researchers to use high resolution remote sensing data. Among them QuickBird, SPOT, IKONOS and aerial photography data were very useful. Pasqualini [5] used SPOT 5 data to map the distribution of *Posidonia oceanica* along Zakinthos Island in Greece with Principal Component Analysis, obtained good results and the accuracy was between 73%-96%. Fornes [6] applied IKONOS data to detect *Posidonia oceanica* in the Mediterranean Sea and found that seagrass distributed 15 m underwater can be detected clearly. Another study also done in the Mediterranean Sea with SPOT and IKONOS showed that four classes: low seagrass cover, high seagrass cover, superficial mobile sediments and deep mobile sediments, can be detected [7]. By comparison with Quickbird, Landsat-5 and CASI data, the conclusion was reached that mapping of seagrass cover, species and biomass to high accuracy levels (> 80%) was not possible across all image types [8]. Hernández-Cruz *et al.* [9] applied documented aerial photography to compare seagrass variation from 1937 to 2000, and regarded that seagrass increase could be described well by a 2^nd^ order polynomial function.

High turbidity is usually a problem for seagrass detection with remote sensing. Phinn [10] retrieved the seagrass data along the coast of Moreton Bay, Australia and found that seagrass in turbid waters was relatively difficult to detect. In order to obtain better results by remote sensing, a water correction was necessary when seagrass was distributed in a submerged state. Considering the absorption and backscattering of CDOM, suspended solids, and chlorophyll a, algorithms were developed to detect seagrass with high accuracy. Hashim [11] studied the seagrass distribution along the coast of Malasia in the south coast of South China Sea using radiation transfer theory, and obtained good results. Mini-distance methods were used for studying the seagrass variation in Izembek Lagoon (Alaska) from 1987 to 1995, and it was concluded that the seagrass area varied little [12].

In China, large area of seagrass have only been found along the South China Sea coast; however, few studies on seagrass distribution change in China have been conducted. In order to protect coastal environments, seagrass distribution change and the reasons behind the changes were always a key problem for seagrass management and protection. In this paper, *in situ* hyperspectral measurements and three types of remote sensing images (Landsat, QuickBird and CBERS) were used to detect seagrass distribution changes in Xincun Bay, Hainan Province. The first aim was to find out how seagrass distribution changed in Xincun Bay from 1991 to 2006; and the second was to give a regional bio-optical model of seagrass for detecting seagrass distribution with high accuracy; the third was to provide the information of seagrass distribution and living state in the area for the government and people to understand how to preserve and protect seagrass.

## 2. Sites Description

**Figure 1.**
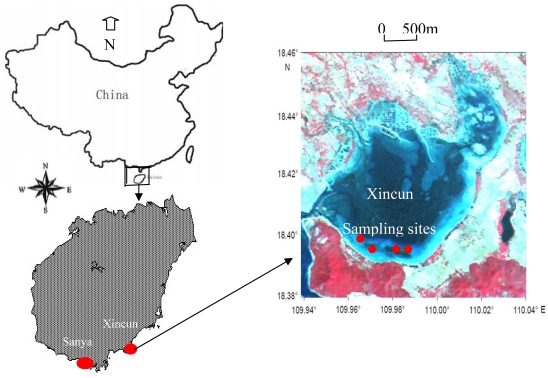
Study area in Xincun Bay, Hainan Province, China.

Xincun Bay (Figure 1), an almost closed bay with only one narrow tunnel connected with the open sea to the southwest, is located in the southeast of Hainan Province; it has an area of more than 13.1 Km^2^ and was famous for its pearl and fish aquaculture, with an aqua-cultural area of more than 52,500 m^2^ and a total fish production of 1,105 tons every year.

Four species of seagrass (*Enhalus acoroides, Thalassia hemperichii, Cymodocea rotundata and Halodule uninervis*) were found distributed in Xinchun Bay, with *Enhalus acoroides* and *Thalassia hemperichii* being the dominant species [13]. On the south coast of Xincun Bay, seagrass distribution was dense and continuous; on the northeast coast, seagrass was only spottily distributed. Water quality deteriorated in recent years from the frequent human activities, leading to algal bloom that appeared in 2003. In order to protect the water environment, Xincun Bay was designated as a marine reserve for seagrass by the government of Hainan Province in 2007 to protect the seagrass ecosystem.

Sediments in Xincun bay can be divided into coarse sand type, coarse-middle sand type, middle sand type, middle-fine sand type, fine sand type and clay type. Among them, fine sand -clay type are mainly distributed along the south coast of Xincun Bay.

The average water temperature in Xincun Bay is 26.25°C in October and 30°C in March [14]. Salinity is 33.74 ‰ in whole bay on average [14], which does not vary greatly for there is no large rivers and only two small streams flowing in.

Xincun Bay is an area that frequently affected by typhoons, with two typhoons on average passing through every year, but even though the area was frequently affected by typhoons and open sea hydrodynamics, seagrasses grew densely and continuously around the south coast of Xincun Bay.

In order to obtain a bio-optical model for high accuracy seagrass remote sensing, field observations were also made in Sanya Bay. Sanya Bay, located in the south of Hainan Province, to the west of Xincun Bay, with location of 109°20′∼109°30′E, 18°11′∼18°18′N and an area of 120 Km^2^, contains coral coasts, mangrove coasts, sand coasts, rocky coasts and seagrass coasts [15]. Sanya Bay is an open bay, with fresh water from the Sanya River flowing in. According to the results of Yang's study [13], seagrass was distributed with high density along the coast of Sanya Bay in 1970s. However, presently seagrass is distributed sparsely due to the frequent anthropogenic activities.

## 3. Material and Methods

### 3.1. In situ Seagrass Observation

**Figure 2.**
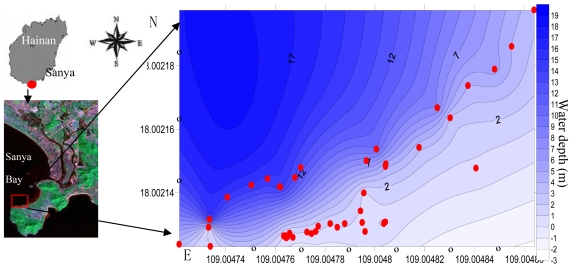
Experiments carried out in Sanya Bay for the training algorithm.

*In situ* observation of seagrass in the coastal area of Xincun Bay was made in April, July, October 2005, January 2006 [15] and November 2007. In order to train the algorithm obtained from Xincun Bay, further field experiments were made in Sanya Bay in April 2008 (Figure 2). Seagrass density, leaf length, leaf area indexes, stems biomass, above ground biomass and water depths were measured. Seagrass density was obtained by counting seagrass number in a 1 × 1 m white square frame from photos. Leaf length and width was measured by ruler *in situ*. Stem biomass and above ground biomass were obtained as weight before (wet weight) and after (dry weight) drying at 60°C in an oven. Seagrass distributed in sub-tidal areas was observed with a self-made underwater video system.

Leaf area index (LAI) can be computed from the accumulation of seagrass leaf density (*μ*) and average plant height (*Z*). Seagrass was relatively short and one side area methods for terrestrial vegetation are unsuitable. This can be described as:

(1) LAI=μ⋅Z

Seagrass density (*D*) can be computed as number of seagrass (*N*) in specific area (*S*), and can be described as the following equation:

(2) D=N/S

Sediment classification was by sampling *in situ* and observation under magnification. Water depths were measured by a rope with an iron weight on one end.

### 3.2. Hyperspectral Measurement and Analyzing

*In situ* spectral measurement was used for obtaining the algorithm used by satellite remote sensing. Spectral reflectance refers to the ratio of the detected radiance reflected from a target surface to the total incidence irradiance. Remote sensing reflectance measurements in this project were made using USB-4000 miniature fiber optical spectrometer, which is made in ocean optic company, USA. Surface spectral measurement was made by using USB-4000 miniature fiber optical spectrometer and profile measurement of *E_u_* and *E_d_* was made by using a water proof modification of the USB-4000 miniature fiber optical spectrometer. Two sensors were measured in the same step.

Lee's bio-optical model [16, 17] and Maritorena and Leathers' algorithm [18, 19] were tentatively used when seagrass was covered with water:

(3) $$r_{rs}={r_{rs}}^{C}+{r_{rs}}^{B}$$

(4) $$R_{rs}\approx \frac{0.5r_{rs}}{1-1.5r_{rs}}$$

(5) $$A={r_{rs}}^{C}+(r_{rs}-{r_{rs}}^{C})\cdot \exp(-2K_{d}H)$$

In the calibration process *r_rs_^C^* was regarded as the reflectivity without bottom reflectance, for $R_{}=\frac{E_{u}}{E_{d}}$, *r_rs_^C^* can be calculated by the following equation:

(6) $${\left(\frac{1-{r_{rs}}^{C}}{1+{r_{rs}}^{C}}\right)}^{2}=\frac{{\left[E_{d}(z)-E_{u}(z)\right]}^{2}}{{\left[E_{d}(z)+E_{u}(z)\right]}^{2}}$$

where *R_rs_* is remote sensing reflectance; *r_rs_* is remote sensing reflectance just below the surface; *r_rs_^C^* and *r_rs_^B^* are the signals from water column and bottom, respectively; *K_d_* is attenuation coefficient of downwelling irradiance; *H* is the bottom depth; *E_u_* is upwelling irradiance and *E_d_* is downwelling irradiance. *E_u_* and *E_d_* can be measured *in situ*.

The Normalized Difference Vegetation Index (NDVI) is a simple numerical indicator that can be used to analyze remote sensing measurements. In order to fully explore the useful information in hyperspectra, the red band reflectance of NDVI was replaced by the green and blue band reflectance. The equations are listed as follows:

(7) Red NDVIRNDVI=(NIR‐Red)/(NIR+Red)

(8) Green NDVIGNDVI=(NIR‐Green)/(NIR+Green)

(9) Blue NDVIBNDVI=(NIR‐Blue)/(NIR+Blue)

where, NIR is near infrared reflectance. In the paper, VNDVI is taken as the general name of BNDVI, GNDVI and RNDVI.

### 3.3. Satellite Data and Processing

#### 3.3.1. Satellite Data

**Figure 3.**
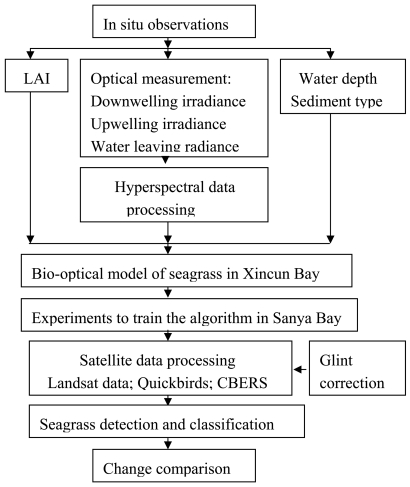
Flow chart of data processing for seagrass retrieval.

China Brazil Earth Resources Satellite (CBERS), Landsat TM and QuickBird data were used in this paper. QuickBird data (2006-1-22) with 4 m (multi-band) spatial resolution, purchased from the Space Eye Company, was used. Landsat TM (1991-10-30; 1999-12-31; 2001-10-1) with 30 m spatial resolution was downloaded from the Global Land Cover facility. China Brazil Earth Resources Satellite data (CBERS, 2006-1-12) with 20 m spatial resolution was provided by the Chinese Remote Sensing Centre. Consdering the band set of satellite images, QuickBird, Landsat TM and CBERS had almost the same bands in the visible and infrared wavelengths; these bands were Band 1 (0.45-0.52 μm), 2 (0.52-0.59 μm), 3 (0.63-0.69 μm) and 4 (0.77-0.89 μm). Bands 1, 2, 3 and 4 of these three sets of satellite data were used to retrieve seagrass distribution in the south coast of Xincun Bay, Hainan Province. Cloud coverage on CBERS, Landsat TM in 1991, 1999 and Quickbird was zero; however, cloud coverage on Landsat TM in 2001 was at 8% or so. Fortunately, there was no cloud cover on the study area. When satellite data was applied to detecting seagrass distribution, the algorithm retrieved with *in situ* observations were used directly. The data processing flow chart for seagrass retrieval is shown in Figure 3.

#### 3.3.2. Glint Correction

Sun glint is usually noise when substrate information is retrieved. The effect of sun glint may be reduced or removed by considering that the near-infrared radiance is practically totally absorbed in the water, thus the observed radiance can be considered to have an origin either as reflected from the surface or as scattered in the atmosphere. In order to obtain better results, an infrared band around 750 nm was used to determine the surface reflectance. The correction was done using the spectral distribution of the extra-terrestrial radiance, although it would be considered more correct to use downwelling radiance measured at the surface.

#### 3.3.3. Image Classification

ENVI and Photoshop software were used to process the satellite images and chlorophyll a was found to very useful for seagrass detection, as the pigments in seagrass were mainly chlorophyll a, chlorophyll b and anthocyanin, and among them chlorophyll a was the dominant pigment. In this paper, regional sediment type mapping was not used, for the sediments in Xincun Bay are mainly sands. Seagrass can be easily differentiated from sediments with satellite remote sensing as their reflectivity differed greatly, though the types of sand sediments are relatively different.

The Normalized Difference Vegetation Index (NDVI) was used to detect seagrass information and false color were used. Different substrates can be differentiated by DN (digital number) range, seagrass leaf area index (LAI) and coverage (under 20%, 20-40%, 40-60%, 60-80% and greater than 80%) which were used as indexes for seagrass classification. Five classes of seagrass distribution were classified with QuickBird, however, only seagrass distribution contour can be retrieved with Landsat TM and CBERS. The difficulty is that the DN of the same substrate in different images varied greatly. In order to make an accurate classification, typical ground objects, such as sand on the south coast of Xincun Bay, were used to normalize the DN of the whole image. Seagrass classification accuracy was calculated by comparing the pixels of satellite remote sensing data with *in situ* observations. For each location a score of the match was assessed and sum of the scores was normalized as the detection accuracy [4].

## 4. Results

### 4.1. Relationship between Seagrass LAI, NDVI and Hyperspectral Bands

Hyperspectra, leaf area index and NDVI (Normalized Difference Vegetation Index) of seagrass were measured and calculated with equations (1, 7, 8, 9), and the relationship between them was obtained.

**Figure 4.**
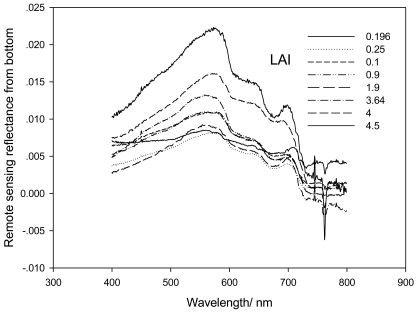
Relationship between spectral bands and seagrass LAI.

As Figure 4 indicates, the spectral reflectance of seagrass measured at different stations changed regularly with LAI. The bands with a good correspondence with LAI were 555, 635, 650 and 675 nm, for absorption and reflectance of seagrass photosynthetic and accessory pigment. However, at the band around 400 and 720 nm, a relatively poor relationship with LAI was found (Table 1). The peaks and troughs on the reflectance spectra were also affected by factors such as reflectance and transmission of single leaves, types of background, leaf angle, the geometry of sun and sensor angles, etc, and these effects can be reduced to great extent when hyperspectral measurement are taken *in situ*.

**Figure 5.**
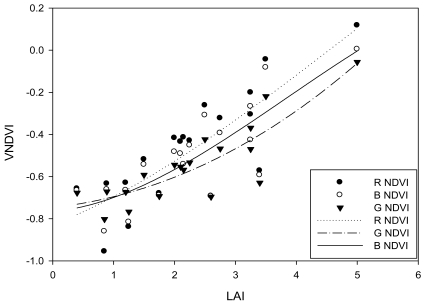
Relationship between LAI and NDVI.

In order to monitor seagrass with satellite remote sensing, the relationship between Leaf Area Index (LAI) and the Normalized Difference Vegetation Index (NDVI) was correlated. Analysis indicated that a good relationship existed between NDVI and LAI. Relationships between every VNDVI (RNDVI, GNDVI and BNDVI) and LAI were studied. Figure 5 shows that the VNDVI increased with the increase of LAI. The correlation coefficient between G-NDVI and LAI (0.7357) was better than the RNDVI (0.6705) and BNDVI (0.6729), which means that GNDVI is more sensitive than RNDVI and BNDVI when applied to seagrass remote sensing. However, when LAI is less than 1.5, the correlation coefficient between VNDVI and LAI is relatively low.

As the band set in the visible and infrared of the satellite remote sensing data (CBERS, Landsat TM, and QuickBird) is blue (0.45-0.52μm), green (0.52-0.59μm), red (0.63-0.69μm) and infra-red (0.77-0.89μm), NDVI was retrieved with blue, green and red also, which is compatible with the satellite data band set.

### 4.2. Seagrass Detection with Satellite Remote Sensing

**Figure 6.**
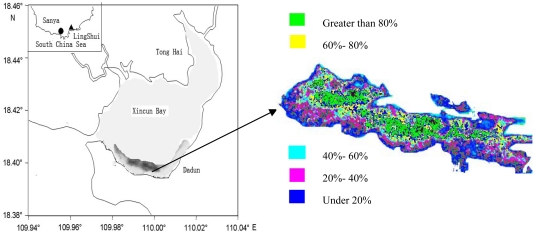
Seagrass density retrieved with QuickBird.

QuickBird data was used for correcting seagrass density and detailed distribution retrieved with Landsat and CBERS data, for detailed seagrass distribution was very important for us to compare seagrass distribution changes on the south coast of Xincun Bay. With the computation of band math (Green NDVI), seagrass distribution along the south coast of Xincun Bay was retrieved and is shown in Figure 6. From the QuickBird image, substrate types, such as sand, seagrass, etc. can be detected clearly, and the profiles from bank to the centre of Xincun Bay were sea pond, sand, seagrass and optically deep water. Seagrass distribution can be easily detected from the evident spectral differences of the background.

The pattern of seagrass distribution can also be clearly classified, and seagrass was mainly distributed in a stripe pattern, some tens of meters away from the coastline. Seagrass density is regular in the main seagrass bed. From the outside to the centre of the main seagrass bed, seagrass distribution coverage was under 20%, 20-40%, 40-60%, 60-80% and greater than 80%. Among them, the area of seagrass coverage greater than 80% accounted for more than 30% of the total seagrass bed (Figure 6). Seagrass species in Xincun Bay are mainly *Enhalus acoroides, Thalassia hemprichii, Cymodocea rotundata and Halodule uninervis*; however, we could not differentiate one species from another with QuickBird data.

Detection accuracy of seagrass with Quickbird data was mainly by comparing pixels of satellite remote sensing with *in situ* observations. In this paper, the accuracy was more than 80% for seagrass coverage greater than 20% when compared with the *in situ* observation results.

Detailed information on seagrass distribution was very important for us to know the density of seagrass distribution and can be used as basis for comparing seagrass distribution changes. In order to further study seagrass distribution changes in Xincun Bay, Landsat TM and CBERS data was used. Compared with QuickBird, seagrass detected with Landsat TM and CBERS had fewer classes, which only showed the distribution range, for pattern and species cannot be clearly obtained. However, the distribution contour can be detected clearly, which was enough for comparison of seagrass distribution changes.

**Figure 7.**
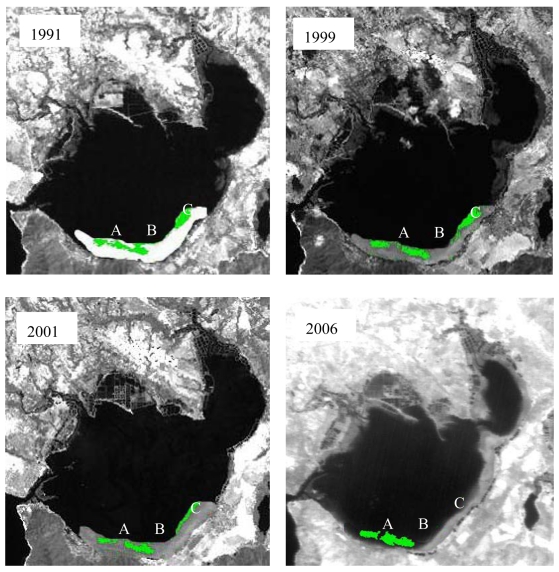
Seagrass distribution change from 1991 to 2006.

Seagrass distribution in the south coast of Xincun Bay was mainly studied. In order to compare seagrass distribution in detail, we divided seagrass distribution regions as A, B and C (Figure 7). Seagrass distribution in region A and region B was connected as one big seagrass bed in 1991, however, the two region seagrass separated gradually and they were only connected with a line of seagrass in 1999. Finally, complete separation was observed in 2001.

Seagrass distribution in region C was relatively large with an elliptical shape in 1991; however, the shape of seagrass bed became thinner by 1999 and became a line in 2001. In 2006, seagrass at region C could be detected with satellite remote sensing.

## 5. Discussion

Xincun Bay is a relatively better place for comparison of seagrass distribution change with satellite remote sensing, for seasonal variations were not evident, and satellite remote sensing data in different seasons can be used for seagrass change comparisons (satellite images of late winter and early spring were used in this paper).

Seagrass distributed on the south coast of Xincun Bay, Hainan Province, are mainly *Enhalus acoroides, Thalassia hemperichii*. Spectral contributions of seagrass differed greatly when it was distributed in different environments, such as distribution in different water depths and confunded with other substrates. When seagrass is distributed underwater, remote sensing reflectance was affected by the water body and water column correction was necessary. Equations of water color remote sensing can be used for water column correction [16, 20].

The relationship between seagrass leaf area index (LAI) and hyperspectra is very important when satellite remote sensing data is applied for detecting seagrass distribution. However, the multi-band set in satellite data were not compatible with hyperspectral bands and the best algorithm retrieved with hyperspectral remote sensing should be adapted when applied to seagrass remote sensing. As the link between LAI and satellite remote sensing, NDVI of blue, green and red band was used in this paper, for multi-band set of the satellite data (CBERS, Landsat TM, and QuickBird) is blue, green, red and infra-red.

The accuracy of seagrass remote sensing can be affected by many factors. Among them the main ones are image resolution, quality and algorithm, water clarity, depth of seagrass distribution and seagrass density. In order to improve classification accuracy, *in situ* observation and high resolution Quickbird images were used as a correction for seagrass detection with low resolution remote sensing data. Background was also an important factor, affecting seagrass detection accuracy, fortunately, sediments in Xincun Bay were mainly sand, which makes it relatively easier to detect seagrass by remote sensing.

Seagrass distributed on the south coast of Xincun bay was preserved very well before 2001, though the shape of the seagrass bed varied, mainly because Xincun Bay is a relatively closed bay, with only one narrow channel connecting it to the open sea, and any hydrodynamic power produced by tides and strong winds was relatively weak. Gong, *et al.* [21] studied the hydrodynamics of Xincun Bay with *in situ* measured data and 1-D hydrodynamic models, and showed that the small inlets attenuated the power of hydrodynamics when tides occurred. However, a great change occurred in the seagrass distribution after 2001; and seagrass could not even be detected in region C in 2006. The reasons for this seagrass reduction were mainly human activities, especially land use change in recent years, and extreme natural accidents, such as typhoons and extreme tidal hydrodynamics.

**Figure 8.**
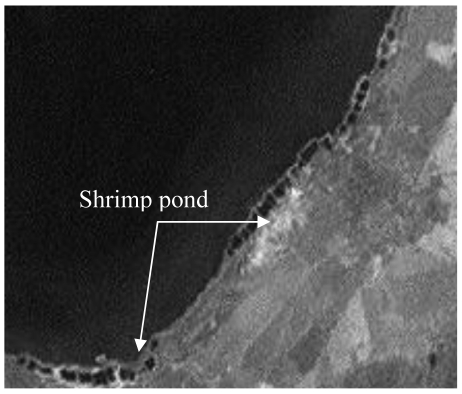
Shrimp ponds around Xincun Bay in 2001.

Human activities, such as construction of shrimp ponds, aquaculture, fishing with standing net, clam digging, boat sailing, capturing prawns and fishes with blasting and trawling, affected seagrass growth in shallow waters. The area dedicated to shrimp ponds increased greatly in recent years, which had great negative effects on seagrass distribution (Figure 8).

**Figure 9.**
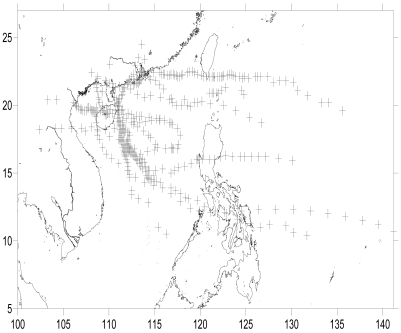
Pathway of typhoons affecting Xincun Bay since 2000.

Xincun Bay is an area where two typhoons pass by every year on average, so typhoons are another important factor that influences seagrass distribution in Xincun Bay (Figure 9). As typhoon pass by, direct physical damage, resuspended sediments and sediment-containing run off, affected seagrass growth, especially for land use change in recent years (the more area of land use change the less area of seagrass).

Tidal hydrodynamics and transparency is also important factors that affected seagrass distribution in Xincun Bay. Tidal hydrodynamics affected sediments (mainly sand) close to the waterline, on which seagrass cannot flourish. Light is also a very important factor for seagrass growth, and clear shallow water is mainly distributed in the south of Xincun Bay, where it provided suitable conditions for large seagrass growth areas to flourish. However, in recent years, water quality has deteriorated.

## 6. Conclusions

Seagrass distribution in Xincun Bay spanning 15 years (1991-2006) was retrieved with satellite remote sensing. From the seagrass detection results, the resolution of satellite remote sensing image is very important for seagrass detection, so QuickBird data was more suitable for seagrass detection than Landsat TM and CBERS, especially when the seagrass distribution area was relatively small. Results in the paper proved that five classes can be classified clearly with QuickBird; however, only seagrass distribution contours can be detected with Landsat TM and CBERS data.

Though the accuracy of seagrass detection with satellite remote sensing can be affected by many factors, seagrass in Xincun Bay can be detected clearly for the sediment there was sand. Compared with satellite remote sensing data in 1991, the seagrass distribution area was reduced gradually and large areas of seagrass had disappeared by 2006. The reasons for seagrass distribution changes were also analyzed in the paper. Human activities and extreme natural disasters were the main reasons for seagrass reduction, especially land use changes in recent years. In a word, seagrass recovery cannot recover from these disturbances.

## Figures and Tables

**Table 1.** Relationship between LAI and different hyperspectral band.

550 nm	y = 0.0011x^2^ - 0.0026x + 0.0099	R^2^ = 0.8846
635 nm	y = 0.001x^2^ - 0.0028x + 0.0072	R^2^ = 0.8575
650 nm	y = 0.001x^2^ - 0.0029x + 0.0068	R^2^ = 0.8512
675 nm	y = 0.0008x^2^ - 0.0026x + 0.0053	R^2^ = 0.7929
400 nm	y = 0.0011x^2^ - 0.0041x + 0.0068	R^2^ = 0.7728
700 nm	y = 0.0008x^2^ - 0.0024x + 0.0057	R^2^ = 0.7604

In Table 1, y is remote sensing reflectivity from bottom, x is LAI
